# A retrospective study of provisional outcomes of intracorporeal esophagojejunostomy versus extracorporeal anastomosis during laparoscopic total gastrectomy for gastric cancer. a single -center

**DOI:** 10.1186/s12957-024-03548-6

**Published:** 2024-12-03

**Authors:** Maladho Tanta Diallo, Zhao Shuai, Bangquan Chen, Yantao Yu, Zhang Yan, Qiannan Sun, Daorong Wang

**Affiliations:** 1 Northern Jiangsu People’s Hospital Affiliated to Yangzhou University, Yangzhou, 225001 China; 2 General Surgery Institute of Yangzhou, Yangzhou University, Yangzhou, 225001 China; 3 Medical College of Yangzhou University, Yangzhou, 225001 China; 4 Yangzhou Key Laboratory of Basic and Clinical Transformation of Digestive and Metabolic Diseases, Yangzhou, 225001 China

**Keywords:** Gastric Cancer, Intracorporeal, Extracorporeal, Esophagojejunostomy; Surgery, Laparoscopy, Total Gastrectomy

## Abstract

Gastric cancer (GC) treatment is increasingly undergoing laparoscopic total gastrectomy (LTG) procedures. However, we conducted this research to evaluate postoperative outcomes, particularly surgical complications associated with intracorporeal and extracorporeal esophagojejunostomies using linear or circular stapling techniques following LTG for GC treatment. We aimed to compare short-term postoperative outcomes, such as surgical complications and anastomotic outcomes, between the two groups.

Method

From January 2020 to August 2022, we conducted a retrospective analysis of data from 160 consecutive patients diagnosed with GC who received either IEJ (*n* = 35) or EEJ (*n* = 125) during LTG. We utilized the Mann–Whitney U test to ascertain the statistical significance between the two groups. For comparing categorical variables, including numbers and percentages, we employed either the Pearson chi-square test, continuity correction, or Fisher's exact test as appropriate.

Results

The operative time was similar (IEJ: 184.57 ± 36.489 vs. EEJ: 189.22 ± 43.584; *P* = 0.565), however, the number of positive lymph nodes was performed more in the IEJ group (IEJ: 4.71 ± 6.114 vs. EEJ: 6.39 ± 9.067 (*P* = 0.305). The blood loss in the IEJ group was lower than that of the EEJ (IEJ: 73.1429.182 vs. EEJ: 100.6461.693 mL, *P* = 0.012). There were three anastomosis leakages in the EEJ and one in the IEJ group (EEJ, 3.2% vs. IEJ, 2.8%; *P* > 0.999). Anastomosis bleeding only occurred in the EEJ (EEJ 1%; *P* = 0.003). Although the EEJ linear stapling technique had two deaths (EEJ, 1.6%).

Conclusion

Although EEJ is frequently utilized in the linear stapling technique, research indicates that the use of IEJ minimizes the incidence of complications in LTG.

**Supplementary Information:**

The online version contains supplementary material available at 10.1186/s12957-024-03548-6.

## Introduction

Gastric cancer (GC) is a global prevalent malignancy affecting both men and women. It ranks as the fifth most frequently diagnosed cancer and the fourth leading cause of cancer-related mortality. In 2020, an estimated 769,000 new cases and deaths were reported, with China representing almost half of the total cases and fatalities [1]. Although, the major cause of GC is unknown; however, several factors including a complicated interaction between the host and the environment, with Helicobacter pylori being a prominent risk factor [2], dietary factors, tobacco consumption, low socioeconomic status, and a family history of the disease are various risk which can influence the genetic cell changes in your stomach [3]. In China, due to factors like the absence of a large-scale endoscopic screening program and integrating of new technologies the disease remains mainly at an advanced stage [4]. The framework emphasized the need for early diagnosis and treatment to facilitate cancer prognosis and reduce cancer-specific mortality [5].

With the recent advancement in GC treatment like molecular targeted and chemotherapy therapy, total gastrectomy has adopted as an effective surgery preferably, with minimally invasive for postoperative outcomes. Numerous advantages such as less pain, better cosmetic results, faster recovery, and shorter hospital stays than open surgery due to its smaller (8- to 10-cm) midline incision, predictive severe complication and rick factors to patience has been discussed in a meta-analysis by Kim.et.al [6]. Extracorporeal esophagojejunostomy (EEJ) in laparoscopic total gastrectomy (LTG) is conducted in a manner akin to the conventional esophagojejunostomy in open total gastrectomy, which was previously in use. Typically, following the total removal of the lymph nodes and stomach, the trocar incision is enlarged to facilitate the extraction of the esophageal stump and jejunum, allowing for the extracorporeal completion of the esophagojejunostomy. Similarly, the challenges associated with esophagojejunal anastomosis require a significant level of laparoscopic surgical proficiency. A mini-laparotomy's restricted and deep operating space presents challenges for both anvil insertion and esophagojejunal anastomosis, often leading to difficulties or ambiguity. Intracorporeal esophagojejunostomy (IEJ) circumvents the need for mini-laparotomy and offers a superior surgical view compared to EEJ, albeit necessitating more advanced skills. The intracorporeal esophagojejunostomy techniques (IEJ) demonstrate favorable surgical outcomes, including comparable rates of anastomotic complications [7]. With the linear stapling technique, IEJ requires more skill than EEJ, but it avoids the necessity for a mini-laparotomy and offers a superior operational perspective. Thus, with the benefits of a magnified operative view and direct viewing of the entire anastomosis procedure, intracorporeal anastomosis is regarded as a safe and useful surgical approach. Certain investigations have shown the short-term surgical results of different kinds of intracorporeal anastomosis in smaller retrospective studies, showing a positive outcome [8, 9]. Hence, the potential decrease in surgical complications resulting from intracorporeal anastomosis is currently unclear. Moreover, there is a scarcity of reported findings from randomized studies and reviews that specifically investigate the outcomes of IEJ and EEJ after LTG. This study aims to assess the feasibility and safety of both IEJ and EEJ procedures in the context of LTG.

## Materials and methods

### Ethical standards

Data for this study were obtained from the Surgical Gastric Cancer Patient Registry at Northern Jiangsu People's Hospital in Yangzhou City, Jiangsu Province, P.R. China. The medical records of hospital visitors were reviewed to collect the necessary information. The establishment of this database, concerning the medical records within the GC database, was approved by the Northern Jiangsu People's Hospital Research Ethics Committee. Before analysis, patient data underwent deidentification and anonymization processes.

#### Study population

**Fig. 1:**
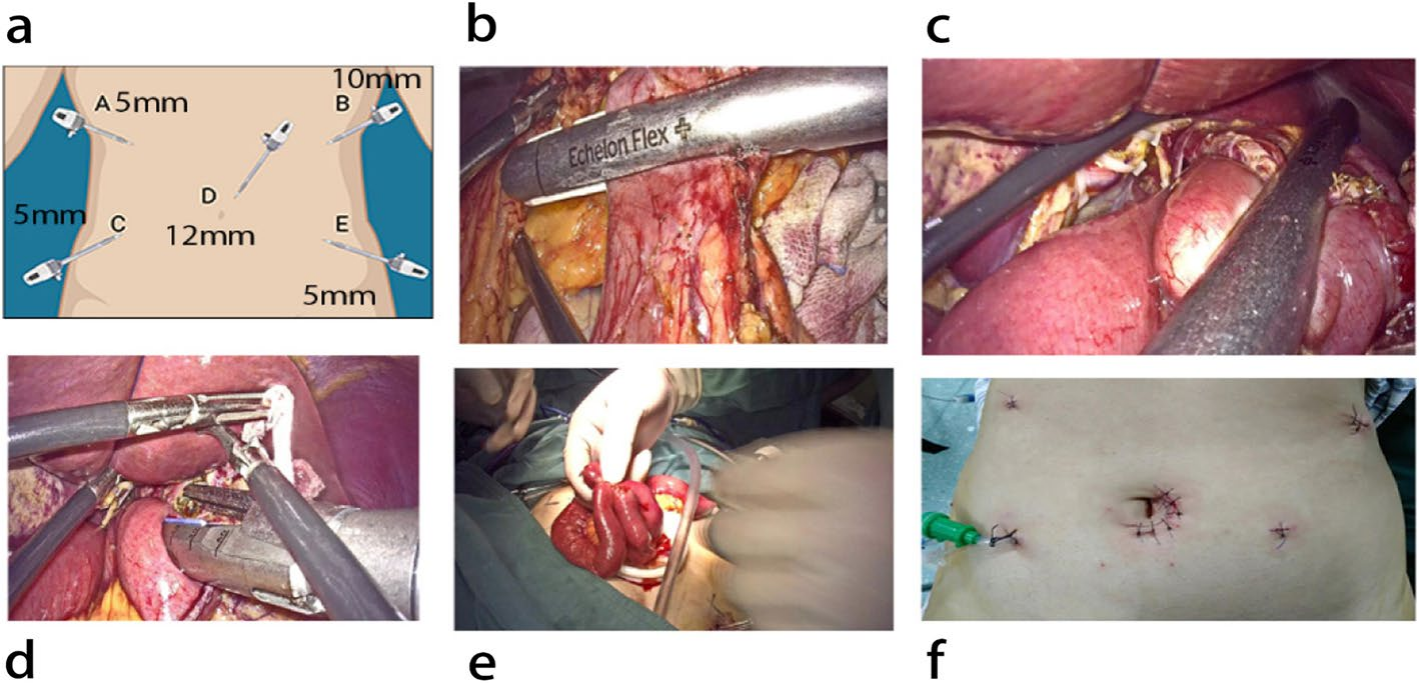
**a** Illustrations of intracorporeal images, surgical diagrams, (**b**) aligning the severed duodenum in an isoperistaltic manner, (**c**) anastomosis is created via a linear cutting stapler, (**d**) suturing the common opening intracorporeal, (**e**) lateral anastomosis of the small intestine, (**f**) placement of trocars for exploration of the abdominal cavity

The data gathered from a retrospective cohort study carried out in the Department of Gastrointestinal Surgery at Northern Jiangsu People's Hospital between January 2020 and August 2022 was collected and utilized. The inclusion criteria are as follows: (1) All patients ages were from 18 to 85; (2) patients were histologically confirmed with gastric adenocarcinoma by biopsy and gastric cancer or Siewert type II or III esophagogastric junction cancer; (3) patients who underwent radical resection (pathologically confirmed R0 resections); (4) patients who underwent D2 lymph node dissection and esophagojejunum completion via a circular anastomosis or cavity through an assisted incision; internal anastomosis (including Roux-en Y anastomosis); (5) patients who completed 6-month follow-up and complete clinical and follow-up data (6) patients were chosen according to the final pathologic result and based on inclusion criteria. The exclusion criteria included: (1) patients with other gastric neoplasms; (2) patients with Siewert's type I esophagogastric junction carcinoma; (3) patients with gastric remnant cancer; (4) patients who underwent a distal or proximal gastrectomy alone without a D1 + /D2/D2 + lymphadenectomy; (5) patients with peritoneal dissemination and other distant organ metastasis; (6) incomplete case data (8) patients with lost or incomplete check-up data. Other criteria were selected based on the surgeons; thus, they should have more experience performing open gastrectomy procedures and laparoscopic gastrectomy procedures. The specific methods of EEJ with the linear stapling technique and IEJ (Fig. 1) were chosen based on the surgeon's preference.

A total of 160 patients were selected based on the selection criteria, of whom 35 underwent IEJ, and 125 underwent EEJ with the linear stapling technique during the LTG of GC, according to the Japanese Gastric Cancer Association's (JGCA) guideline [10]. We decided to detect the difference between IEJ (the newest technique) and EEJ (the old technique) to demonstrate which was more effective with less morbidity and mortality. According to the Japanese Gastric Cancer Association's (JGCA) 14th edition treatment guideline, the amount of lymphadenectomy was categorized [10]. The 7th edition of the Union for International Cancer Control TNM (UICC) staging system was used to determine the tumor's stage [11]. The categorization of postoperative morbidity into either local or systemic morbidity was conducted utilizing the Clavien-Dindo classification system [12].

#### Surgical procedure

Prior to the procedure, all participants chosen for our study underwent standard CT scans of the abdomen and chest to assess the clinical stage of the tumor and identify any additional operative abnormalities. Each patient was placed in a supine position on the operating table, with multiple operative trocars positioned accordingly (Fig. 1). The dissection of local lymph nodes and the mobility of the stomach were similar between the two groups. Following exploration of the abdominal cavity, a complete gastrectomy ensued.

#### Intracorporeal group

Five locations for operational trocars were used: (A) 5 mm trocar, (B) 10 mm trocar, (C) 5 mm trocar, (D) 12 mm trocar and (E) 5 mm. The camera port was inserted through the 12 mm trocar into the infra-umbilical region. Using camera visualization, a pneumoperitoneum of 12 mmHg was established to guide the placement of the remaining four ports. The first trocar was positioned in the umbilical region (left midclavicular line, in the left paraumbilical area, about one handbreadth from the camera port). A 5 mm auxiliary operating port was then placed on the patient's left side (rib margin, left anterior axillary line, at the level of the umbilicus, and left midclavicular line) to assist the surgeon during the operation. This port facilitated the insertion of an endo-stapler for resection of the duodenum, stomach, or abdominal esophagus, as well as the placement of gauze or a suction device to clear the operative field (Fig. 1a).

After transecting the distal esophagus with a linear stapler, the entire stomach was removed. An experienced anesthetist transorally inserted the OrVil anvil until the tip of the transoral tube reached the staple line at the esophageal stump. Using a harmonic scalpel, a small hole was created at the left edge of the staple line, and the tube was exteriorized through this hole until the center rod of the anvil was visible with the aid of a laparoscopic grasper. After releasing the thread connecting the transoral tube and the anvil, the tube was extracted from the abdominal cavity through a trocar hole, completing the anvil insertion. The jejunum was then transected 15–20 cm distal to the Treitz ligament using a linear stapler. Following the removal of the specimen collection bag, the circular stapler was introduced into the jejunal limb through an opening near the cutting margin. A silk string was used to create a slipknot, anchoring the jejunal loop to the stapler shaft and the center rod to prevent the loop from slipping and tearing the surrounding tissues. The jejunal stump was closed with a linear stapler, and on-table endoscopy was routinely performed to evaluate the anastomosis (Through the left upper port wound, a side-to-side jejunojejunostomy was performed using a linear stapler to construct the Roux-en-Y limb (Fig. 1.b, c, d, e, f) (see video supplementary file). All surgeries were conducted by specialized surgeons with significant experience in performing GC surgeries.

#### Extracorporeal group

Following the dissection of the local lymph nodes, a small midline incision was made in the epigastric region, proportionate to the tumor size. The incision was shielded, and the field of vision improved using a wound protector. The distal esophagus was secured with purse-string forceps, and the proximal esophagus was fitted with the anvil. Through a mini laparotomy, the specimen and jejunum were extracted. A circular stapler was then used to perform a standard extracorporeal Roux-en-Y esophagojejunostomy. Finally, a Roux-en-Y limb was created by performing a side-to-side jejunojejunostomy using a linear stapler.

#### Clinicopathologic materials

We utilized Clavien-Dindo's classification system to categorize postoperative complications, while the JGCA was employed to categorize clinicopathologic aspects. Clinicopathologic characteristics and surgical outcomes, including age, sex, body mass index (BMI), tumor location, extent of lymphadenectomy, pathologic stage, number of harvested lymph nodes, overall operation time, estimated blood loss, intraoperative complications, days of liquid food, days of drainage, postoperative hospital stay, postoperative morbidity, and mortality within 30 days of operation, were reviewed.

#### Statistical analysis

Statistical analysis was performed employing either the student’s t-test or the appropriate Mann–Whitney test. Fisher's exact test, or the Chi-square test, was utilized for qualitative data evaluation. A two-tailed P-value of 0.05 (*P* < 0.05) or lower was considered statistically significant. Quantitative data were presented with standard deviations (± SD). All data were analyzed using the SPSS 13.0 software (IBM Corp., Armonk, NY, USA).

## Results

### Patients sex characteristics

#### Patients' characteristics

Table 1 provides a summary of the patients' baseline characteristics. The investigation revealed no statistically significant variances in age, BMI, smoking, alcohol consumption, or the presence of chronic conditions such as (high blood pressure, diabetes, and coronary heart disease) between the extracorporeal and intracorporeal groups.

**Table 1:** Characteristics of patients at baseline

Characteristics	Intracorporeal (*n* = 35)	Extracorporeal (*n* = 125)	*P* value
Age (year, Mean ± SD)	65.57 ± 6.674	66.7 ± 8.256	0.460
BMI (kg/m2, Mean ± SD)	22.54 ± 2.439	23.16 ± 2.674	0.218
Smoking (n [%])			0.227
Yes	11(31.42%)	27(21.60%)	
No	24 (68.57%)	98(78.40%)	
Alcohol (n [%])			0.182
Yes	8(22.85%)	17(13.6%)	
No	27(77.14%)	108(86.4%)	
High blood pressure (n [%])			0.878
Yes	11 (31.43%)	41 (32.80%)	
No	24 (68.57%)	84 (67.20%)	
Diabetes (n [%])			0.510
Yes	3 (8.57%)	5 (4.00%)	
No	32 (91.43%)	120 (96.00%)	
Coronary heart disease (n [%])			1.000
Yes	0 (0.00%)	2 (1.60%)	
NO	35 (100.00%)	123 (98.40%)	

*SD* Standard deviation, *BMI* Body mass index

### Intraoperative and postoperative results

Table 2. The intraoperative and early postoperative results of the EEJ and IEJ groups are shown in Table 2**.** Although the operative durations were similar between the IEJ group (189.22 ± 43.58 mn) and the EEJ group (184.57 ± 36.49 mn) (*P* = 0.565), a notable contrast was observed in average blood loss, with the IEJ group exhibiting lower blood loss compared to the EEJ group (73.14 ± 29.18 mL vs. 100.64 ± 61.69 mL, *P* = 0.012)**.** Short-term outcomes such as time to first flatulence, time to first liquid diet, and postoperative hospital stay show no significant differences between the IEJ and EEJ groups using the linear stapling technique. However, the surgical cost per patient was considerably higher in the EEJ group compared to the IEJ group (64,407.43 RM/yuan vs. 51,653.44 RM/yuan). **NB:** IEJ and EEJhospitalization costs were 51653.44 RM/yuan and 64407.43 RM/yuan per patient, respectively.

**Table 2:** Intraoperative and postoperative results

Variables	Intracorporeal (*n* = 35)	Extracorporeal (*n* = 125)	*P* value
Operative time (min, Mean ± SD)	184.57 ± 36.489	189.22 ± 43.584	0.565
Blood loss (mL, Mean ± SD)	73.14 ± 29.182	100.64 ± 61.693	0.012
Postoperative hospital stays (day, Mean ± SD)	14.91 ± 5.527	16.23 ± 5.640	0.222
Days of drainage (day, Mean ± SD)	2.80 ± 0.677	3.00 ± 0.635	0.200
Days of liquid food (day, Mean ± SD)	2.94 ± 0.639	3.12 ± 0.630	0.177

**NB:** Intracorporeal esophagojejunostomy and extracorporeal esophagojejunostomy hospitalization costs were 51,653.44 RM/yuan and 64,407.43 RM/yuan per patient, respectively

### Surgical outcomes

Table 3. The surgical specimens' pathological analysis was not statistically significant between the IEJ and EEJ with the linear stapling technique groups in terms of pathological tumor stage. In terms of pathological examination, the IEJ group exhibited lower pT and pN stages compared to the EEJ group. The vessel carcinoma embolus in the IEJ and EEJ groups were numerically different, but no significant difference (10 (28.57%) vs. 50 (40%), respectively, was noted. The mean number of lymph nodes and the number of positive lymph nodes in the IEJ group and EEJ group were different, and statistical significance was not observed 26.17 ± 9.718, 23.88 ± 9.860 (*P* = 0.225), and 4.71 ± 6.114, 6.39 ± 9.067 (*P* = 0.305), respectively.

**Table 3:** Surgical outcomes

Variables	Intracorporeal (*n* = 35)	Extracorporeal (*n* = 125)	*P* value
Pathological tumor stage (n [%])			
T			0.160
T1	3 (8.57%)	15 (12.00%)	
T2	7 (20%)	12 (9.60%)	
T3	8 (22.58%)	18 (14.40%)	
T4	17 (48.57%)	80 (64.00%)	
N			0.889
N0	13 (37.14%)	41 (32.80%)	
N1	5 (14.29%)	21 (16.80%)	
N2	7 (20.00%)	21 (16.80%)	
N3	10 (28.57%)	42 (33.60%)	
M			1.000
M0	35 (100.00%)	124 (99.20%)	
M1	0 (0.00%)	1 (0.80%)	
Stage ([%])			0.690
I	8 (22.86%)	21 (16.80%)	
II	9 (25.71%)	29 (23.20%)	
III	18 (51.43%)	74 (59.20%)	
IV	0 (0.00%)	1 (0.80%)	
([%])			0.981
Yes	18 (51.43%)	64 (51.20%)	
No	17 (48.57%)	61 (48.80%)	
Vessel carcinoma embolus (n [%])			0.217
Yes	10 (28.57%)	50 (40%)	
No	25 (71.43%)	75 (60%)	
Number of lymph nodes (Mean ± SD)	26.17 ± 9.718	23.88 ± 9.860	0.225
Number of positive lymph nodes (Mean ± SD)	4.71 ± 6.114	6.39 ± 9.067	0.305

### Intraoperative and postoperative complications

Table 4**.** The study observed no statistically significant differences in terms of overall rate, severity, or incidence of mild problems (Clavien Grade 2) between the groups undergoing intracorporeal esophagojejunostomy and extracorporeal esophagojejunostomy. There were two deaths (1.6%) and 30 days of mortality. Overall morbidity in the IEJ group and the EEJ group was 1 (2.86%) vs. 6 (4.80%), respectively (*p* = 1.000). On the contrary, the IEJ group exhibited a markedly lower complication rate (2.8% vs. 3.2%, *p* = 1.00) compared to the EEJ group. There was no significant difference in the incidence of grade ≥ grade 3 complications between the two groups. One patient had complications (2.8%) in the IEJ group (one anastomosis leakage), and in the EEJ with the linear stapling technique group, four patients had complications (3.2%) (three anastomosis leakages and one anastomosis bleeding). There was zero (0) case mortality in the IEJ group. In contrast, in the EEJ group, we observed two deaths (1.6%): one patient's death because of anastomosis leakage and a second case of death because of pulmonary embolism in the 30-day postoperative period. The 3 months postoperatively between the two procedures during follow-up were zero (0), and the anastomosis stenos was not observed.

**Table 4:** Complications (Clavein grade) of the patients

Variables	Intracorporeal (*n* = 35)	Extracorporeal (*n* = 125)	*P* value
Complication			1.000
** I**	0	0	
** II-III**	Anastomosis leakage (n = 1) 2.8%	Anastomosis leakage (n = 3) Anastomosis bleeding (n = 1) 3.2%	
** IV-V**	0	Death (n = 2) 1.6%	
** Total (%)**	1 (2.86%)	6 (4.80%)	

## Discussion

**Fig. 2:**
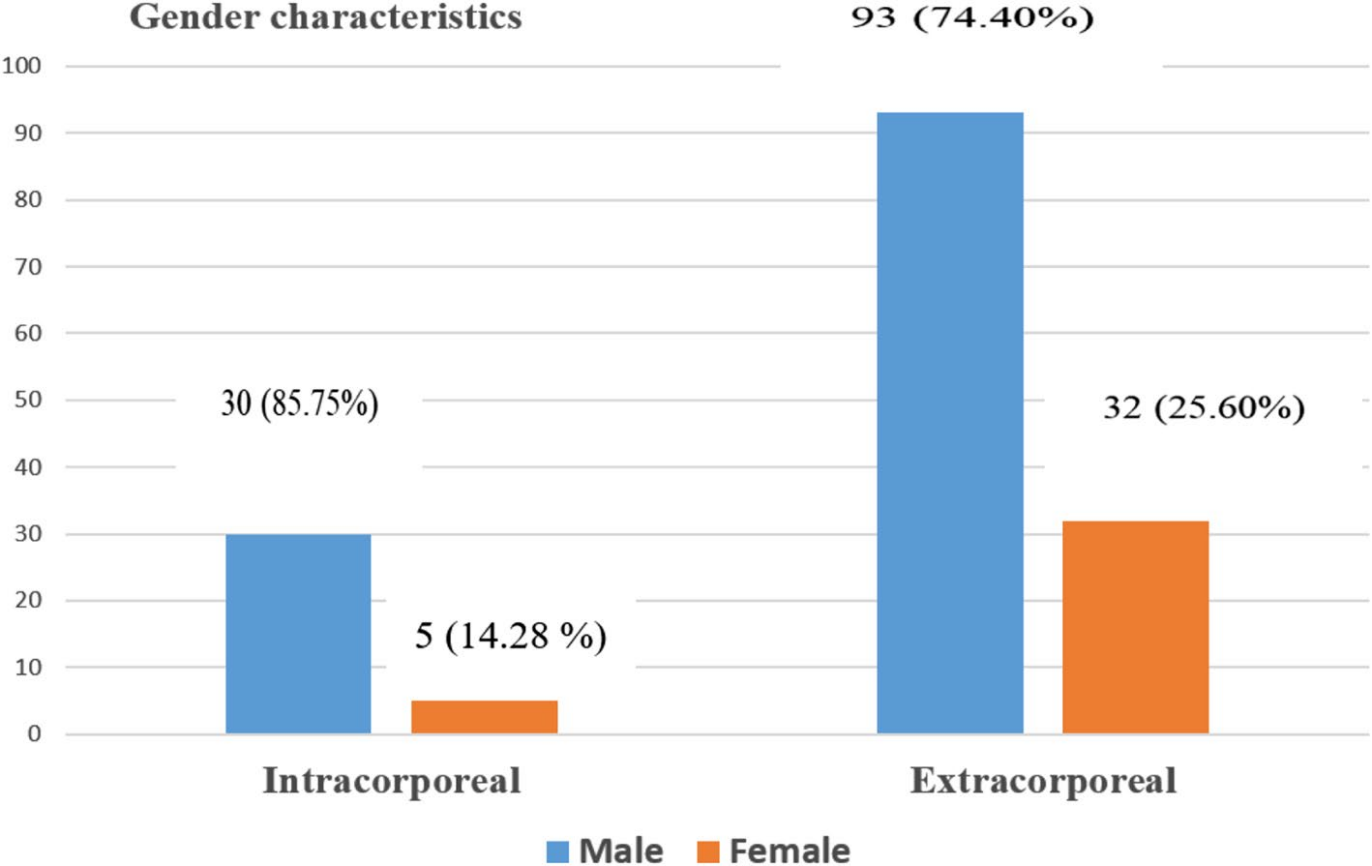
Out of the 160 patients’ data collected for this study, 30 (85.75%) males and 5 (14.28%) females were characterized in the IEJ group, while 93 (74.40%) males and 32 (25.50%) females were also characterized in the EEJ group, respectively. The characteristics of the patients' sexes show that men were more vulnerable than women in both groups (EEJ and IEJ); the EEJ had more male and female patients than the IEJ group

In this research, we demonstrated that surgeons used the EEJ method more commonly than the IEJ one. The number of patients in the IEJ and EEJ groups was 35 and 125, respectively (Fig. 2). In our study, the increased use of EEJ methods could be due to surgeons' difficulties in performing the IEJ method. A similar study was published by Chen et al. [13], indicating that more patients underwent LTG on EEJ (121 patients) than those who used LTG on IEJ (92 patients). Although, in the aforementioned study, the author did not explain the reason for the difference between the two groups. Nonetheless, we suggested that the challenge of IEJ raises questions about surgical safety, which can be explained by the challenges faced by surgeons when using this IEJ technique.

Recently**,** research has shown some innovative techniques and methods for IEJ, like side-to-side anastomosis with liner staplers, functional end-to-end anastomosis with liner staplers, and end-to-side anastomosis with Orvil™ that may be helpful to facilitate surgical procedures [14, 15]. The most crucial component of reconstruction following total gastrectomy is an esophagogastric jejunostomy. The difference between IEJ and EEJ with the linear stapling technique LTG begins with an esophagojejunostomy, which is often performed in a deep, constrictive surgical space. IEJ provides normal anastomosis and always avoids injuries to the surrounding structures. IEJ at LTG is the most difficult and complex method, even for expert surgeons, which is why surgeons use it in comparison to EEJ. IEJ involves a smaller incision and reduces manipulation and operating field exposure. With magnified surgical vision, IEJ is conducted with more care. This study showed that the IEJ had a better postoperative recovery and a safe procedure during LTG for GC.

In our study, we showed that comparatively, the male incidence was higher than the female incidence [93 (74.40%) and 32 (25.60%)] in the EEJ using a linear or circular stapler after group. Similarly, the incidence in males and females was higher than in the IEJ group [30 (85.75%) and 5 (14.28%)]. A similar report by Lu et al. [16] showed that in the IEJ group, the incidence of males was 22 (88%), females 3 (12%), and in the EEJ group, the male incidence was 21 (84%), females 4 (16%). We also found in this study that the number of smokers and alcoholics was higher in the EEJ group than in the IEJ group (Table 1). Studies have shown smoking increases the risk of GC, and many studies have recently shown an increase in smoking and drinking among men. Research indicates that smoking elevates the risk of gastric cancer by 60% in men and by 20% in women compared to those who do not smoke [16, 17]. The present study revealed no statistically significant variances between the two groups concerning baseline characteristics and different chronic diseases such as age, BMI, smoking, alcohol consumption, high blood pressure, diabetes, and coronary heart disease. Shim et al. [18], escribed a similar situation in their study; however, no significant change was detected. According to a recent meta-analysis, laparoscopic distal gastrectomy (LDG) with intracorporeal anastomosis is theoretically viable, safer, and less invasive than extracorporeal anastomosis [20]. Another research study has proven the similar safety and viability of total laparoscopic gastrectomy with IEJ [19]. Another research study has demonstrated total laparoscopic gastrectomy's comparable safety and viability with IEJ [20]. Our observation noted that studies with IEJ were rarely practiced and limited in the Northern Jiangsu People's Hospital. This variation could be attributed to the technical difficulty of the IEJ.

There is limited research comparing the surgical outcomes of intracorporeal and extracorporeal anastomosis after lung transplantation (LTG). However, the majority of studies suggest that intracorporeal anastomosis procedures do not carry an increased risk of anastomosis leakage or other postoperative complications [12]. However, certain studies have suggested enhanced surgical recovery, such as an earlier initiation of diet or a reduced duration of hospitalization [21, 22]. This is most likely a result of the identical perioperative care strategy that was used to manage the two anastomosis groups, including diet [20]. Our study noted that 35 patients underwent IEJ, and 125 underwent EEJ with a linear or circular stapler technique. There was no significant difference in the mean operation time between the two groups. Similar operation time, with a mean blood loss in the IEJ was less than in the EEJ using a linear or circular stapler after group, was statistically significant. Furthermore, there was no difference in the mean length of hospital stays between the IEJ and EEJ groups. According to a study conducted by Jeong et al. [23], the surgical outcomes of LTG with OrVil™ esophagojejunostomy were favorable. However, Jeong et al. [23] indicated one patient had an intraabdominal abscess that required surgical drainage and a mean operation time of 194 min (range 160–270 min). Our results observed statistically significant blood loss between the IEJ and EEJ groups (Table 2). On the contrary, the average duration of the operation was 257 min (range, 212 to 302 min), and the estimated blood loss was 69 ml (range 5 to 187 ml). The mean duration for performing purse-string suturing was 6 min (range 5 to 7 min) [24].

In our study, the IEJ cost was less than the EEJ group. This could be because IEJ has fewer complications and shorter postoperative hospital stays than EEJ with the linear stapling technique. In addition, it’s worth noting that, utilizing the same LTG and lymphadenectomy techniques, both the IEJ and EEJ groups retrieved a similar number of lymph nodes. Nonetheless, no significant differences were identified. In other studies, two patients developed lymph node metastases where the average number of collected lymph nodes was 33 (range, 18–49), and all resected tissues exhibited tumor-free resection margins [24]. On pathological examination, EEJ with the linear stapling technique after collected more nerve invasion and vascular carcinoma embolus cases than IEJ, and no differences were significant between the two groups. In stage IV, no significant differences were observed between the two groups, with only minimal variations detected. On the other hand, the stages were similar in stages I, II, and III (Table 4). In contrast, Kinoshita et al. [24] indicated that all 41 of the patients had tumor-free margins, and the pathological stages were stage I in 10 (24.4%), stage II in 27 (65.9%), and stage III in 4 (9.7%) of the patients.

Studies have indicated that IEJ anastomosis is performed less frequently for LTG because it is technically feasible, safe, and has a quicker postoperative recovery than EEJ with the linear stapling technique. Unfortunately, the majority of prior research did not show the effectiveness of IEJ in decreasing postoperative complications following LTG [25]. In comparison to extracorporeal anastomosis following LTG, our study revealed that IEJ considerably decreased complications, including anastomotic bleeding and deaths. The broad anastomotic lumen, enhanced operative view, and tension-free anastomosis with less tissue damage in intracorporeal anastomosis are most likely to blame for this. Anastomotic leakage can result from insecure anastomosis, leading to severe consequences that can worsen the long-term prognosis, lengthen the hospital stay following surgery, and raise medical expenses. Leakage rates varied from 0% to 16.7% in previously published trials on LTG with OrVil [18, 23].

Our postoperative complications that are related to IEJ and EEJ with the linear stapling technique manifested in the EEJ group differently than in the IEJ group. In this study, the rate of complications was another principal advantage of intracorporeal anastomosis. The occurrence of complications was notably reduced in the IEJ group (2.8% vs. 3.2%, *p* = 1.00) compared to the EEJ group. In the IEJ group, only one patient had anastomosis leakage. Contrary to this, we had several complications, including four patients with complications (3.2%) (three anastomosis leakages and one anastomosis bleeding) in the EEJ group.

In our study, the EEJ group identified a higher rate of complications compared to the IEJ group, likely due to surgical procedures. For instance, on-table endoscopy was not employed to examine the esophagus lumen and direct laparoscopy vision was not utilized to monitor the circumferential wall of the anastomotic site. According to Lu et al. [26], pneumonia and intra-abdominal infections were complications. On the other hand, our results showed no complications following the anastomosis. However, postoperative morbidity was comparable in both groups (28.0% versus 32.0%, P 14 0.758). Contrarily, we found zero (0) case mortality in the IEJ group.

Our study did not find any significant differences in pulmonary complications or quality of life between the IEJ and EEJ methods. This lack of evidence may be attributed to the limited number of participants in our study. It is important to note that the transoral intracorporeal laparoscopic total gastrectomy (TLTG) method, which involves IEJ, does not require a mini-laparotomy in the epigastrium. Consequently, the literature suggests that TLTG, or totally laparoscopic distal gastrectomy (TLDG), procedures lead to fewer pulmonary complications and less postoperative pain compared to laparoscopy-assisted gastrectomy procedures. Notably, pulmonary complications are a significant contributor to postoperative mortality in patients with GC [27, 28]. Several studies have indicated that intracorporeal anastomosis techniques result in lower rates of pulmonary complications when compared to EEJ methods. In a previous meta-analysis comparing laparoscopy-assisted total gastrectomy (LATG) to open total gastrectomy (OTG), it was found that respiratory issues played a minor role in the overall decrease in medical complications associated with LATG [29]. Subsequent retrospective studies focusing on patients aged over 65 revealed minimal differences in the incidence of pulmonary complications between the LATG and OTG patient cohorts [30]. Contrary to these findings, early studies comparing TLTG with LATG predominantly discussed anastomotic challenges and did not thoroughly investigate pulmonary outcomes [31, 32]. Research has consistently shown that reduced pulmonary function is more commonly observed following upper abdominal incisions than lower abdominal surgeries [33, 34].

In contrast, in the EEJ using a linear or circular stapler after group, two deaths (1.6%) were observed: one patient's death was due to anastomosis leakage, and the second case was pulmonary embolism in the 30-day postoperative period. Future studies should incorporate more objective criteria for assessing postoperative recovery to investigate the impact of intracorporeal anastomosis on morbidity and mortality.

Our study described the merits and feasibility of the IEJ and EEJ with the linear stapling technique. Therefore, due to the retrospective nature of this study and its presumed critical selection bias, limitations are preventing us from conclusively determining which technique form is more beneficial. Although the results may offer valuable background data for subsequent prospective research, the statistical power may be compromised due to the difference in sample size and a feature of the retrospective approach. Patients were chosen based on inclusion criteria and pathological results, while the surgeons' choice of anastomosis technique (IEJ or EEJ with the linear stapling technique) was determined by their preference. Nonetheless, the baseline characteristics of the two study groups were substantially comparable. The favorable surgical outcomes observed in the IEJ group might have been influenced by the surgeons' expertise, given that intracorporeal anastomosis involved a more challenging approach. Additionally, IEJ is a relatively novel technique compared to EEJ, which typically employs a linear or circular stapler thereafter.

## Conclusion

EEJ, with the linear stapling technique, and IEJ, which come after LTG, are technically feasible. Based on the short-term postoperative results in the present study, the IEJ technique had less blood than the EEJ group, but the operation time was similar in both groups. In addition, the morbidity and mortality rates in the IEJ group were lower than those in the EEJ group. On the other hand, IEJ can also prevent anastomotic complications and death. As a result, the attainment of a secure laparoscopic esophagojejunostomy remains a daunting task. More high-quality research is required to establish the value of IEJ and choose the best anastomosis technique.

## Supplementary Information

Supplementary Material 1.

## Data Availability

The datasets used and/or analyzed during the current study are available from the corresponding author upon reasonable request.
